# Supplementary material for: Novel Polymorphisms in RAPGEF6 Gene Associated with Egg-Laying Rate in Chinese Jing Hong Chicken using Genome-Wide SNP Scan
Source: Genes (Basel). 2019 May 20;10(5):384. doi: 10.3390/genes10050384 (PMC6562510; doi:10.3390/genes10050384)
Supplement: Supplementary file 1 [file genes-10-00384-s001.pdf]

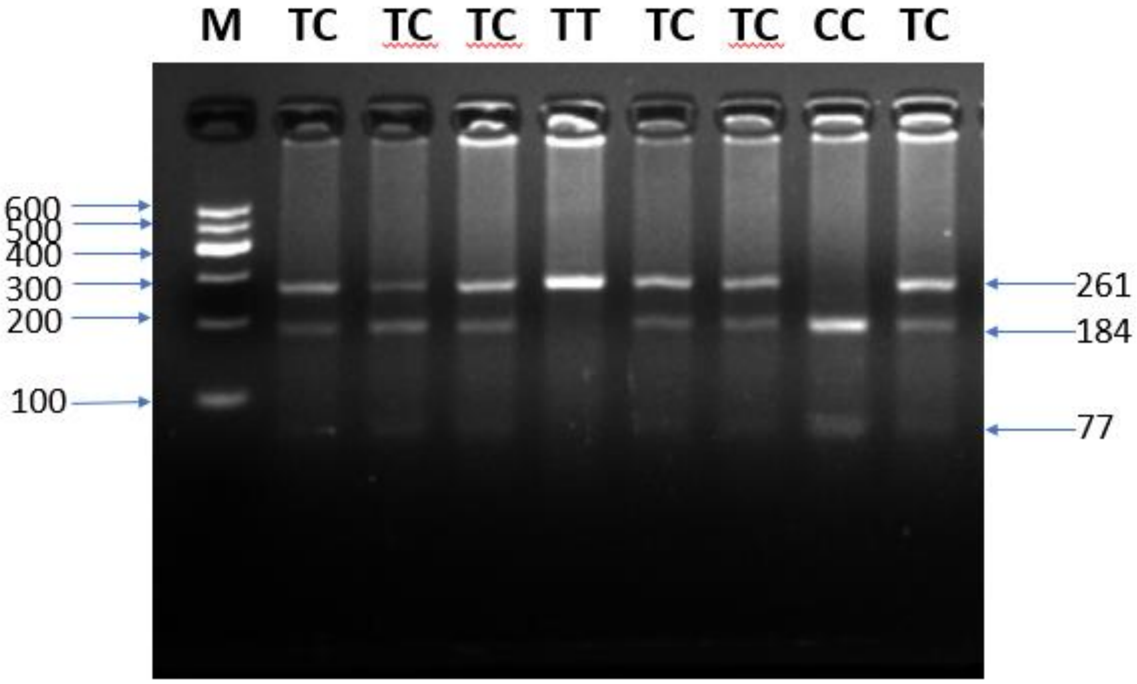

Figure S1. The electrophoretic result of PCR-RFLP band patterns of *RAPGEF6* gene at 15836649 bp in chicken. DNA was digested with the restriction enzyme *MnlI* and three types of genotypes (TT, TC and CC), which are shown above (M: DL600 plus).

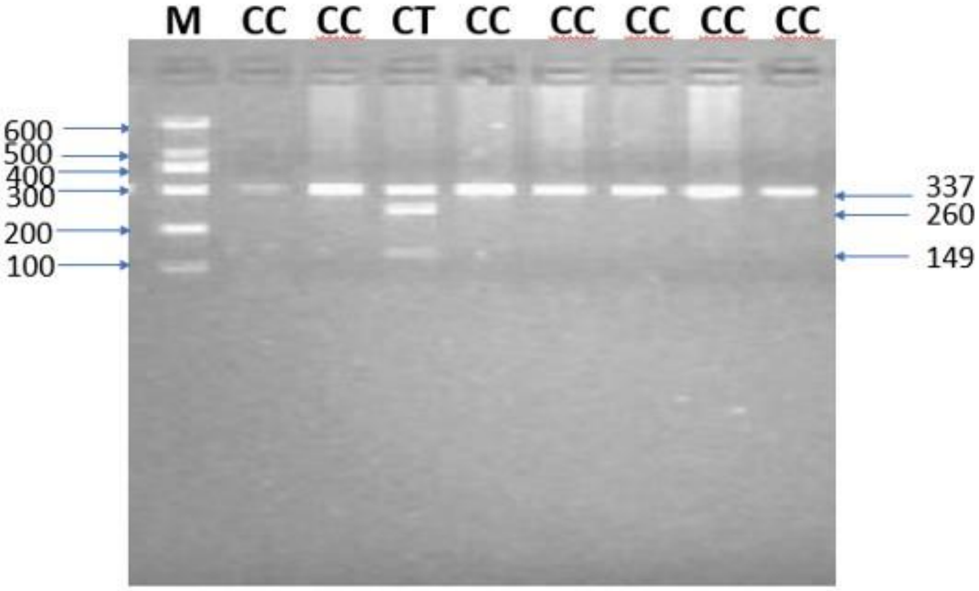

Figure S2. The electrophoretic result of PCR-RFLP band patterns of *RAPGEF6* gene at 15843452 bp in chicken. DNA was digested with the restriction enzyme *MboI* and two types of genotypes (CC and CT), which are shown above (M: DL600 plus).

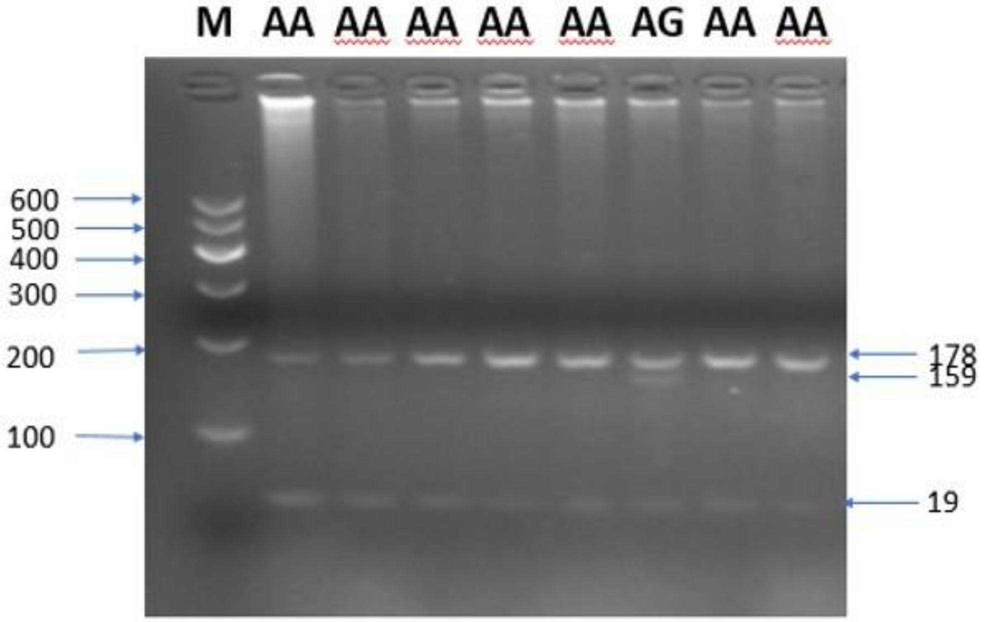

Figure S3. The electrophoretic result of PCR-RFLP band patterns of *RAPGEF6* gene at 15829057 bp in chicken. DNA was digested with the restriction enzyme *XmnI* and two types of genotypes (AA and AG), which are shown above (M: DL600 plus).

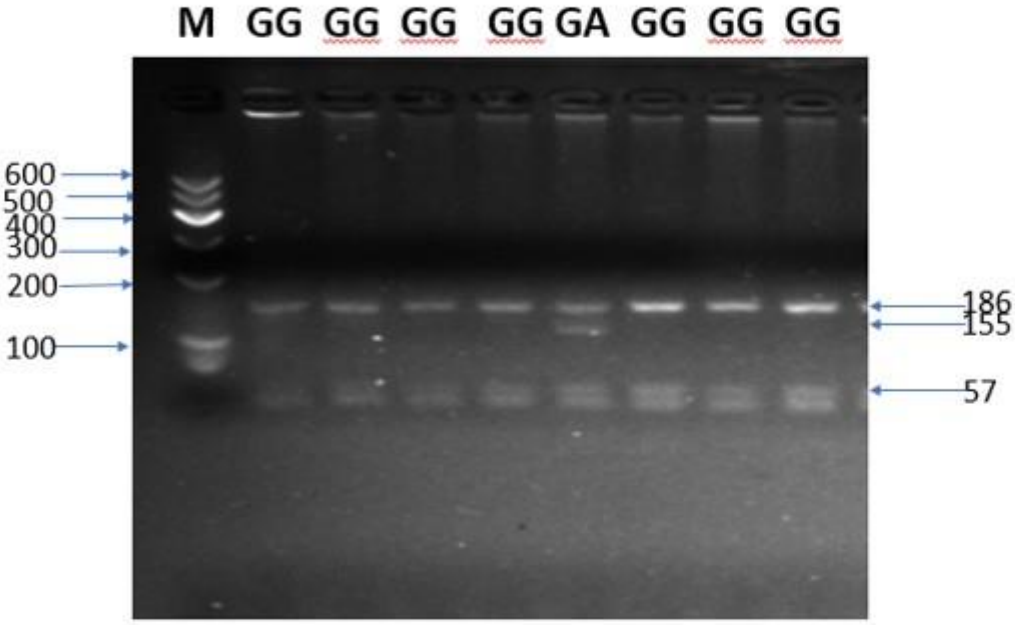

Figure S4. The electrophoretic result of PCR-RFLP band patterns of *RAPGEF6* gene at 15845449 bp in chicken. DNA was digested with the restriction enzyme *MwoI* and two types of genotypes (GG and GA), which are shown above (M: DL600 plus).

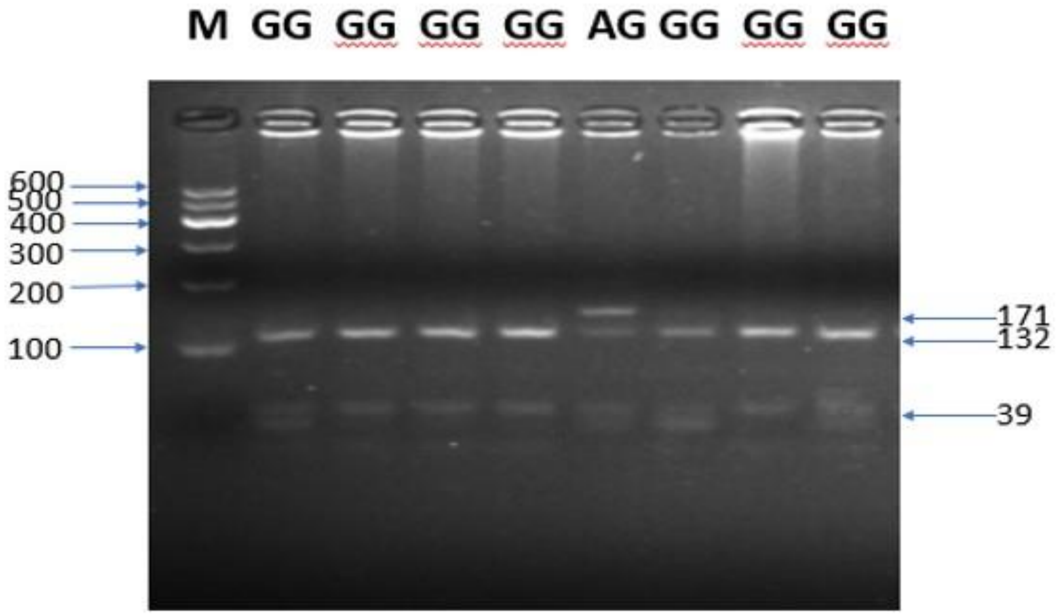

Figure S5. The electrophoretic result of PCR-RFLP band patterns of *RAPGEF6* gene at 15829303 bp in chicken. DNA was digested with the restriction enzyme *HphI* and two types of genotypes (AG and GG), which are shown above (M: DL600 plus).

Supplementary Table S1. Information of five variants (SNPs) in chicken *RAPGEP6* gene.

Sl. No.	Probe Set ID	Affy SNP ID	Position in the genome	Flank	Allele A	Allele B
1	AX-75745366	Affx-50618245	15836649	CCTGTTAGCC[T/C]CCTTAAGAAG	T	C
2	AX-75745380	Affx-50618257	15843452	TCATAACCGA[C/T]CCAAATTTGC	C	T
3	AX-75745340	Affx-50618223	15829057	AGAGACACAG[A/G]AATGAGTGCC	A	G
4	AX-75745388	Affx-50618264	15845449	TATGAGCCAA[G/A]CCATTACTGC	G	A
5	AX-75745341	Affx-50618224	15829303	TAGCAGGATC[A/G]CCTTCAAAGT	A	G

Supplementary Table S2. Primer information for SNP fragments in amplified chicken *RAPGEF6* gene.

Sl. No.	Oligo name	Primers	Sequences (5′ to 3′)	Base	GC%	Product Length	Annealing Temperature(°C)	nmol/OD	MW
1	EGG-A1-F012	Forward	GCACGAATATGGTTTTTCAG	20	40.0	261	54.6	5.1	6147.1
	EGG-A1-R253	Reverse	AGGGCTCCTTGGCTTCCATT	20	55.0			5.7	6075.0
2	EGG-A2-F064	Forward	GGAGGGAGAAGAGCCACTAA	20	55.0	486	53,5	4.7	6249.1
	EGG-A2-R533	Reverse	CTGGACGTCTGGAAAATATG	20	45.0			5.0	6181.1
3	EGG-A3-F148	Forward	CTGTAACACAAGAGACATGAA	21	38.1	178	52.0	4.5	6456.3
	EGG-A3-R305	Reverse	AAAATAGTACACATGGAACTC	21	33.3			4.5	6431.3
4	EGG-A4-F197	Forward	CTCCATTCCTATTTGTACTT	20	35.0	259	53.5	5.7	5984.0
	EGG-A4-R436	Reverse	TAAAATGTAGAAAGCAGGGT	20	35.0			4.6	6238.2
5	EGG-A5-F171	Forward	TTAACCCTTGCAAACACACT	20	40.0	197	50.2	5.4	6005.0
	EGG-A5-R348	Reverse	GCTTTCTTTTCAGGTTACCA	20	40.0			5.5	6049.0

Supplementary Table S3. The ingredients of *RAPGEF6* PCR-RFLP optimized Restriction Enzyme (RE) digestion mixture.

Sl. No.	SNP ID	Restriction Enzyme	Composition of Digestion Mixture (μL)				Digestion Temperature (°C)	% of Agarose Gel
			10 × buffer	PCR products	Enzyme	ddH2O		
1	AX-75745366	*MnlI* (10 U/μL)	1.0	5.0	0.03	4.0	37	5
2	AX-75745380	*MboI* (10 U/μL)	1.0	5.0	0.03	4.0	37	5
3	AX-75745340	*XmnI* (10 U/μL)	1.0	5.0	0.03	4.0	37	5
4	AX-75745388	*MwoI* (10 U/μL)	1.0	5.0	0.03	4.0	60	5
5	AX-75745341	*HphI* (10 U/μL)	1.0	5.0	0.03	4.0	37	5

Supplementary Table S4. The restriction enzymes with their recognition site/cut position used to digest the PCR products.

Sl. No.	Position in VLDLR	Name of Restriction Enzyme	Recognition Sequence	Recognition Site/Cut Position
1	T15835549C	*MnlI*	5' CCTCN_7<br>3' GGAGN_6	5′ CCTC(N)7↓ 3′<br>3′ GGAG(N)6↑ 5′
2	C15843452T	*MboI*	5' GATC<br>3' CTAG	5′ ↓GATC 3′<br>3′ CTAG↑ 5′
3	A15829057G	*XmnI*	5' GAANNNNTTC<br>3' CTTNNNNAAG	5′ GAANN↓NNTTC 3′<br>3′ CTTNN↑NNAAG 5′
4	G15845449A	*MwoI*	5' GCNNNNNNNGC<br>3' CGNNNNNNNCG	5′ GCNNNNN↓NNGC 3′<br>3′ CGNN↑NNNNNCG 5′
5	A15829303G	*HphI*	5' GGTGAN_8<br>3' CCACTN_7	5′ GGTGA (N)8↓ 3′<br>3′ CCACT (N)7↑ 5′
